# Electrically Tunable Multiple‐Effects Synergistic and Boosted Photoelectric Performance in Te/WSe_2_ Mixed‐Dimensional Heterojunction Phototransistors

**DOI:** 10.1002/advs.202400018

**Published:** 2024-03-19

**Authors:** Hechun Cao, Tao Hu, Jiyue Zhang, Dongyang Zhao, Yan Chen, Xudong Wang, Jing Yang, Yuanyuan Zhang, Xiaodong Tang, Wei Bai, Hong Shen, Jianlu Wang, Junhao Chu

**Affiliations:** ^1^ Key Laboratory of Polar Materials and Devices (MOE) and Department of Electronics East China Normal University Shanghai 200241 P. R. China; ^2^ State Key Laboratory of Infrared Physics Shanghai Institute of Technical Physics Chinese Academy of Sciences No.500 Yutian Road Shanghai 200083 P. R. China; ^3^ Shanghai Frontier Base of Intelligent Optoelectronics and Perception Institute of Optoelectronics Fudan University Shanghai 200433 P. R. China; ^4^ Collaborative Innovation Center of Extreme Optics Shanxi University Taiyuan Shanxi 030006 P. R. China; ^5^ Frontier Institute of Chip and System Fudan University Shanghai 200433 P. R. China

**Keywords:** multiple‐mechanisms effects, photodetectors, Te/WSe_2_ heterojunction

## Abstract

Mix‐dimensional heterojunctions (MDHJs) photodetectors (PDs) built from bulk and 2D materials are the research focus to develop hetero‐integrated and multifunctional optoelectronic sensor systems. However, it is still an open issue for achieving multiple effects synergistic characteristics to boost sensitivity and enrich the prospect in artificial bionic systems. Herein, electrically tunable Te/WSe_2_ MDHJs phototransistors are constructed, and an ultralow dark current below 0.1 pA and a large on/off rectification ratio of 10^6^ is achieved. Photoconductive, photovoltaic, and photo‐thermoelectric conversions are simultaneously demonstrated by tuning the gate and bias. By these synergistic effects, responsivity and detectivity respectively reach 13.9 A W^−1^ and 1.37 × 10^12^ Jones with 400 times increment. The Te/WSe_2_ MDHJs PDs can function as artificial bionic visual systems due to the comparable response time to those of the human visual system and the presence of transient positive and negative response signals. This work offers an available strategy for intelligent optoelectronic devices with hetero‐integration and multifunctions.

## Graphical abstract

In this work, electrically tunable multiple‐mechanisms synergistic Te/WSe2 MDHJs phototransistors are constructed. By modulating the gate and bias, the *R* and D* are significantly improved by the synergism of PC, PV, and photo‐thermoelectric (PTE) conversion. Transient PTE photocurrent signals can well simulate the human visual system, implying that the device can undertake the function of artificial bionic visual systems.

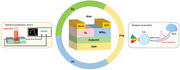

## 1 Introduction

Photodetectors (PDs), the crucial element in an optoelectronic sensor system, convert the light signals to electric ones by photoconductive (PC), photovoltaic (PV), or photo‐thermoelectric (PTE) effect, respectively.^[^ ^1^ ^]^ Usually, the PC PDs show high gain with slow response speed while the PV ones exhibit fast response and self‐powered behaviors with the inferiority of low quantum efficiency. Nevertheless, PTE response intimately depends on the deliberate device construction showing high gain and fast speed. Recently, mix‐dimensional heterojunctions (MDHJs) with bulk semiconductors and 2D materials have been widely built to develop PDs showing superior performance through their unique interfacial effects.^[^ ^2^ ^]^ Moreover, ultrafast photo‐generated carrier transfer and transport, and exciton dissociation are found in MDHJs PDs due to the interfacial effect.^[^ ^3^ ^]^ Intelligent PDs exhibiting low energy consumption, broadband response, and polarized detection are thus demonstrated in MDHJs PDs.^[^ ^3^ ^]^ Thanks to the strong absorption of HgCdTe (MCT) and the anisotropic property of black phosphorus (BP),^[^ ^4^ ^]^ polarization‐sensitive mid‐wave infrared PDs are reported in BP/MCT MDHJs. Broadband Bi_2_Se_3_/GaN MDHJs PDs with an ultrahigh responsivity of 2.45 × 10^4^ A W^−1^ are designed because of the fast electron transport in the surface of Bi_2_Se_3_ and the large built‐in electric field.^[^ ^5^ ^]^ In MDHJs PDs, the configuration dependence on the gate voltage (*V*_GS_) and drain–source bias (*V*_DS_) can induce various responses that promote the transfer and transport of the photogenerated carriers.^[^ ^6^ ^]^ Transient response signals are observed in MoO_3_/Si and SnSe/Si PDs which are attributed to the appearance of the PTE effect where an extra electric field is generated by a photo‐induced temperature gradient at the interface.^[^ ^7^ ^]^ Therefore, MDHJs PDs are the prospect in human vision systems, in‐sensor computing, computing in memory, and neuromorphic devices through the various synergistic effects of PC, PV, and PTE.^[^ ^8^ ^]^

Tellurium (Te) is a typical p‐type semiconductor with high mobility of 1300 cm^2^ V^−1^ s^−1^ showing a strong competitor to BP due to its unique air stability besides the narrow bandgap, thickness‐dependent bandgap, and anisotropic nature.^[^ ^9^ ^]^ Nowadays, various Te‐based MDHJs PDs are constructed to break the limit of insensitive response and low‐temperature operating owing to its narrow bandgap. Te/ReS_2_ MDHJs field‐effect transistors (FETs) are designed where the introduction of Te significantly enhances the absorption and thus the photocurrent.^[^ ^10^ ^]^ Te/MoSe_2_ p–n junctions are fabricated to realize polarization‐sensitive PDs with the anisotropic nature of Te.^[^ ^11^ ^]^ Tunneling Te/In_2_S_3_ heterojunctions with band alignment transfer from type II to type I or III show a high responsivity of 146 A W^−1^.^[^ ^12^ ^]^ More recently, Te/WSe_2_ MDHJs are constructed where a built‐in potential is formed with their different conduction types and thus self‐powered detection is present,^[^ ^13^ ^]^ and electrostatic‐gated reversible rectifying polarity and anisotropic photoresponse is reported with the anisotropic feature of Te.^[^ ^14^ ^]^ Both the conduction type and Seebeck coefficient of WSe_2_ are electrically tuned simultaneously because of their ambipolar nature.^[^ ^15^ ^]^ The photocurrent is controlled according to the appearance of either the photovoltaic (PV) or PTE effect which is determined by the defined local gate configuration.^[^ ^16^ ^]^ Unluckily, the WSe_2_ PDs show a low peak responsivity of 0.7 mA W^−1^ and a short cutoff wavelength only at 800 nm.^[^ ^16^ ^]^ Although self‐powered and anisotropic photoresponse has been detailed,^[^ ^15^ ^]^ electrically tunable multiple‐effects synergistic and enhanced response performance is still absent in Te/WSe_2_ MDHJs along with its ambipolarity and electrically regulated thermoelectric figure of merit of WSe_2_, and the p‐type nature of Te with high concentration.

In this work, Te/WSe_2_ MDHJs built from p‐type Te with high concentration and ambipolar WSe_2_ are fabricated by van der Waals (vdW) integration. An ultralow dark current lower than 0.1 pA and a large on/off rectification ratio of 10^6^ is achieved by energy band alignment engineering. Electrically tunable characteristics significantly improve responsivity and detectivity through multi‐mechanism synergistic effects from PC, PV, and PTE. Peak responsivity and detectivity arrive at 13.9 A W^−1^ and 1.37 × 10^12^ Jones, respectively, with exceeding 400 times increment. The transient photocurrent behaviors and comparable response time of 830 µs to that of the human visual system are demonstrated in Te/WSe_2_ phototransistors, which can well simulate the function of the human visual system suggesting a great potential for artificial bionic vision systems of the Te/WSe_2_ MDHJs.

## 2 Results and Discussions

### 2.1 Device Structure Design and Characterizations

**Figure 1:**
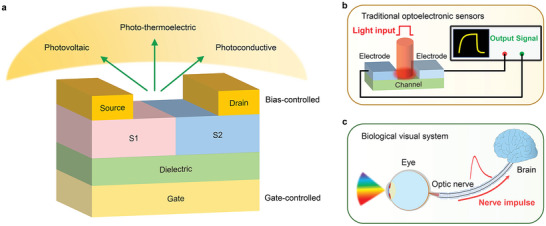
Schematic diagrams of photoelectric mechanisms and visual system. a) Schematic diagram of tunable heterojunction PDs with different optoelectronic conversion effects. b) Schematic diagram of a typical photodetector, where the conversion processes of an optical signal into an electrical signal are demonstrated. c) Schematic diagram of a biological visual system, where optical information is identified and integrated into the eyes (retina) and the visual nerve center in the brain receives information.

The van der Waals interactions break the strict limits of heterostructure integration for different materials, thus making it possible to introduce complex mechanisms in heterogeneously integrated PDs, and further improve and expand the performance.^[^ ^17^ ^]^ In terms of the mechanisms of optoelectronic conversion processes, PDs are categorized as PC, PV, and PTE detectors^[^ ^18^ ^]^ as depicted in **Figure** **1**a. Generally, traditional photon detectors can transfer light signals to electrical signals based on individual PC effects or PV effects, which are mainly used in optoelectronic sensors (Figure 1b). However, the response mechanism of the heterojunction transistor PDs is usually controlled by a bias voltage or a gate voltage.^[^ ^19^ ^]^ The tunable multi‐mechanism synergistic PDs not only endow the typical optoelectronic sensors with more sensitive detection abilities but also provide the possibility of novel artificial bionic visual systems (Figure 1c). The biological visual system is responsible for image sensing and data processing simultaneously.^[^ ^20^ ^]^ The nerve cells in the retina convert the external information into electrical signals and integration information and then transmit the nerve impulse to the brain through the optic nerve (Figure 1c).

**Figure 2:**
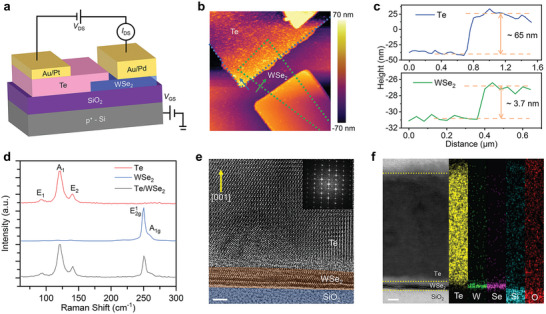
Device structures and characterizations. a) Schematic diagram of the Te/WSe_2_ MDHJs. b) AFM images of the Te/WSe_2_ heterojunction. The areas in the blue dot line and green dot line are Te and WSe_2_, respectively. c) The height profiles of Te and WSe_2_, correspond to the blue and green arrows in Figure 2b. d) The Raman spectra of Te, WSe_2,_ and Te/WSe_2_ MDHJs. e) Cross‐sectional HRTEM image of the Te/WSe_2_ MDHJs on SiO_2_/Si substrate. Scale bar: 2 nm. The inset is the SAED pattern of the transferred Te thin film. f) HAADF‐STEM image and EDS maps of the Te/WSe_2_ MDHJs. Scale bar: 5 nm.

Mixed‐dimensional vdWH PDs with p‐type narrow bandgap Te and ambipolar WSe_2_ are designed and fabricated where multiple optoelectronic response mechanisms are demonstrated through a gate and a bias voltage to enable the prospect in bionic visual systems. Single‐crystalline Te thin films are grown on mica (001) by molecular beam epitaxy (MBE) through a van der Waals epitaxial route^[^ ^21^ ^]^ (Figure S1, Supporting Information). Few‐layer WSe_2_ flakes are exfoliated and transferred on a p‐type heavily doped Si substrate covered with a 300 nm thick SiO_2_ by using a Scotch tape‐based mechanical exfoliation method.^[^ ^22^ ^]^ Polydimethylsiloxane (PDMS) film is spin‐coated on the Te thin films, availing the exfoliation of Te films from the mica substrate and the transfer to the WSe_2_/SiO_2_/Si flakes. **Figure** **2**a shows the 3D schematic diagram of a typical mixed‐dimensional Te/WSe_2_ vdWH device. The contact electrodes of Au/Pt and Au/Pd are fabricated through a mask, electron beam lithography, and ion beam sputtering, which supports a good Ohmic contact with Te and WSe_2_, respectively.^[^ ^14^, ^23^ ^]^ The expected thicknesses of Te and WSe_2_ are also confirmed by an atomic force microscope (AFM) (Figure 2b). Figure 2c displays the height profiles of Te and WSe_2_ with thicknesses of ≈65 and 3.7 nm, respectively.

Figure 2d presents the Raman vibrational spectra of a Te/WSe_2_ MDHJs device at different positions including the individual Te, WSe_2_, and interface area. Three Raman peaks at 92, 121, and 141 cm^−1^ in the red line are in good agreement with the E_1_‐TO, A_1,_ and E_2_ phonon vibrational modes of the hexagonal Te.^[^ ^9d^ ^]^ Raman spectrum of WSe_2_ (blue line) contains a strong peak at 250 cm^−1^ and a weak peak at 260 cm^−1^, which are assigned to be E^1^_2 g_ and A_1g_ vibrational modes, respectively.^[^ ^24^ ^]^ All of these Raman peaks representing both the two materials are found in the overlapped region (black line), indicating the formation of the Te/WSe_2_ MDHJs after the mechanical exfoliation and target‐transfer processes. High‐resolution transmission electron microscopy (HRTEM) is conducted to characterize the details of the Te/WSe_2_ MDHJ interface. An atomically flat interface of Te/WSe_2_ MDHJ without any bubbles or impurities is formed (Figure 2e). The inset shows the Bragg diffraction spots of the selected area–area electron diffraction (SAED) pattern, and the well‐defined SAED illustrates that the exfoliated and transferred Te films maintain high crystal quality and further confirm the out‐of‐plane growth orientation along [001]. High‐angle annular dark field scanning transmission electron microscopy (HAADF‐STEM) and the energy dispersive spectra (EDS) mapping. These imply that the individual elements of Te, W, Se, Si, and O are evenly present in the corresponding layers (Figure 2f), further confirming an atomic interface of Te and WSe_2_.

### 2.2 Electrical and Photoelectric Properties of the Te/WSe_2_ Devices

**Figure 3:**
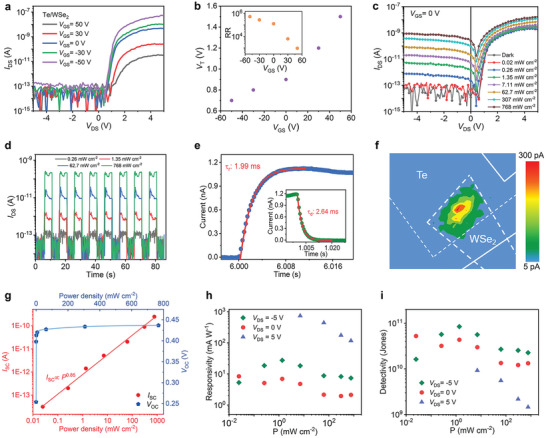
Electrical and optoelectronic properties. a) Output characteristics at different *V*_GS_ from −50 to 50 V. b) The turn‐on voltage and rectification ratio (inset) dependence on the *V*_GS_. c) Output curves in the dark and under a 638 nm laser illumination with various light power densities at *V*_GS_ = 0 V. d) Photoswitching response under a 638 nm laser illumination with different incident powers at *V*_DS_ = 0 V. e) Rise time and decay (inset) time of the photocurrent under a 638 nm laser illumination at *V*_DS_ = 0 V. f) Scanning photocurrent mapping under a 638 nm laser illumination at *V*_DS_ = 0 V. g) The dependence of short‐circuit current and open‐circuit voltage on the light power. h, i) The dependence of *R* and D* on the light power at *V*_DS_ = −5, 0, and 5 V.

The transfer curves of individual Te FETs and WSe_2_ FETs are characterized to confirm their carrier type, respectively. A typical p‐type for Te and an ambipolar nature for WSe_2_ are demonstrated as expected (Figure S2a,b, Supporting Information). Therefore, the Te/WSe_2_ PDs are homologous heterojunctions in an equilibrium state, i.e., p^+^p heterojunction. Moreover, the transfer curve of Te/WSe_2_ MDHJs FETs also shows an ambipolar transport behavior, implying that the WSe_2_ dominates the transport characteristics of the device channel (Figure S2c, Supporting Information). **Figure** **3**a exhibits the *I*_DS_–*V*_DS_ curves of the Te/WSe_2_ MDHJs FETs. The Te/WSe_2_ FET device shows a strong rectification behavior, implying a well‐defined interface and availing the self‐powered detectivity of the Te/WSe_2_ MDHJs PDs.^[^ ^25^ ^]^ Besides, the device has an ultralow reverse current lower than 0.1 pA due to the large built‐in barrier between Te and WSe_2_. Note that the forward output characteristic curves are significantly dependent on a *V*_GS_. The on/off rectification ratio (RR) is strongly modulated by a *V*_GS_ (the inset of Figure 3b) and can arrive at ≈10^6^ when a negative *V*_GS_ of −50 V is applied with more than three orders of magnitude increase. The turn‐on voltage (*V*_T_) is also significantly tuned, which increases from 0.7 to 1.5 V with increasing the *V*_GS_ from −50 to +50 V. These data evidence a strong gate‐tunable current rectifying and switching behaviors of the Te/WSe_2_ MDHJs device, which is superior to those of the other homologous heterojunctions of Te/MoTe_2_^[^ ^26^ ^]^ and MCT/BP.^[^ ^4^ ^]^

Figure 3c illustrates the *I*_DS_–*V*_DS_ curves of the Te/WSe_2_ MDHJs PDs in dark and under various illumination intensities of a 638 nm laser from 0.02 to 768 mW cm^−2^ at *V*_GS_ = 0 V. Obvious photocurrents induced by a PV effect are demonstrated, and the ratio of photocurrent to dark current is beyond 10^4^ at zero bias (Figure 3d), revealing a high signal‐to‐noise ratio and a self‐powered photodetection behavior. The response time including the rise and decay time (*τ*_r_ and *τ*_d_) are measured and determined to be 1.99 and 2.64 ms (Figure 3e), respectively. The response speed is faster than similar Te‐based heterojunction PDs of Te/MoTe_2_ (τ_r_ = 4.8 ms) ^[^ ^26^ ^]^ Te/Bi_2_Se_3_ (τ_r_ = 10 ms),^[^ ^27^ ^]^ and Te/WS_2_ (τ_r_ = 25 ms).^[^ ^28^ ^]^ To confirm the photoresponse origin of the Te/WSe_2_ MDHJs PDs, the scanning photocurrent microscopy mapping was conducted (Figure 3f). The photocurrent intensively occurs at the junction interface area and no other current is generated near the metal contact areas, which explicitly evidences that the PV effect originates from the Te/WSe_2_ heterojunction rather than the metal–semiconductor contact areas. Figure 3g plots that the short‐circuit current (*I*_SC_) has a linear power‐law dependence with incident power (*I*_SC_ ∝ *P* ^0.85^), whereas the open‐circuit voltage (*V*_OC_) displays a logarithmic relation with light intensity and reaches a saturation point of 0.43 V. The above data implies a strong built‐in electric field (*E*_bi_) is formed at the interface of the Te/WSe_2_ p^+^p junction. The device operates on the principle of a p^+^p junction with a unilateral depletion region. This is because there is a hole‐accumulated region on the Te side, while a hole‐depleted region exists on the WSe_2_ side.

For a photodetector, responsivity (*R*) and detectivity (D*) are crucial figures of merit to evaluate their response performance.^[^ ^2^, ^19^, ^22^ ^]^ *R* is calculated by *R* = (*I*_light_−*I*_dark_)/(*P*_in_ *A*_eff_), where the dark current is assigned to the main contribution of noise.^[^ ^22^, ^29^ ^]^ Here, *I*_light_, *I*_dark_, *P*_in,_ and *A*_eff_ are photocurrent, dark current, incident light power density, and effective illumination area of a photodetector, respectively. D* is defined as follows, D* = *R* / (2*eI*_dark_ / *A*_eff_)^1/2^, where *e* represents the electron charge.^[^ ^22^, ^30^ ^]^ Figure 3h,i summarizes the light power dependence of the *R* and D* of our Te/WSe_2_ MDHJs PDs at a bias of *V*_DS_ = −5, 0, and 5 V, respectively. It is found that the *R* and D* are almost independent of the light power when *V*_DS_ at −5 or 0 V while their values decrease obviously with the increasing light intensity when *V*_DS_ is 5 V. To further elucidate the two different behaviors, the light intensity dependence on the photocurrent (*I*_ph_ = *I*_light_−*I*_dark_) is extracted (in Figure S3, Supporting Information), and well satisfies power‐law fitting, *I*_ph_ = A *P^k^*, where A is a constant, *P* is incident power density and *k* is the linearity power exponent. The *k* values are 0.85, 0.97, and 0.61 with *V*_DS_ of −5, 0, and 5 V, which implies that different response mechanisms might occur in the Te/WSe_2_ MDHJs PDs. When *V*_DS_ is −5 or 0 V, the exponent *k* close to the ideal linearity of unit 1 indicates that the PV effect dominates the response.^[^ ^31^ ^]^ While *V*_DS_ is 5 V, the *k* value of 0.61 deviating from the ideal 1 indicates that the detection mechanism converts into PC one.^[^ ^31^ ^]^ When the PDs devices operate at the PV mode, the peak *R* and D* values are determined to be 27.5 mA W^−1^ and 8.43 × 10^10^ Jones at an incident light intensity of 1.36 mW cm^−2^. These values of *R* and D* are comparable to those of the PV detectors of MoSe_2_/WSe_2_,^[^ ^6a^ ^]^ Te/CsPbBr_3_,^[^ ^32^ ^]^ and Te/ZnO.^[^ ^33^ ^]^

### 2.3 Electrically Tunable Multiple‐Effects Synergistic and Enhanced Photoelectric Response

**Figure 4:**
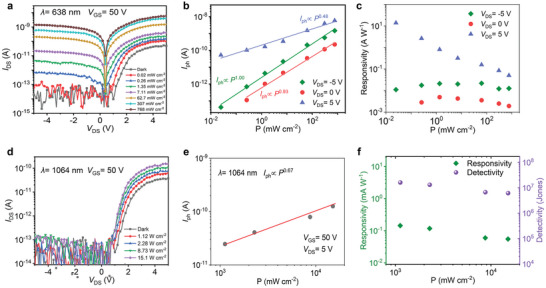
Gate‐modulated photoelectric properties. a) Output curves in the dark case and under a 638 nm laser illumination with various power densities at *V*_GS_ = 50 V. b) Power dependence of *I*_ph_ at *V*_DS_ = −5, 0, and 5 V. c) Power dependence of the *R* at *V*_DS_ = −5, 0 and 5 V. d) Output curves in the darkness and under a 1064 nm laser illumination with various power densities at *V*_GS_ = 50 V. e) Power dependence of the *I*_ph_ under the 1064 nm illumination at *V*_DS_ = 5 V. f) Power dependence of the *R* and D* under a 1064 nm laser illumination.

Photoresponse performance of the Te/WSe_2_ MDHJs PDs modulated by a *V*_GS_ and/or a *V*_DS_ voltage is further investigated. Here, *V*_GS_ = 50 V and *V*_GS_ = −50 V are respectively selected as a typical case, and *I*_DS_–*V*_DS_ curves at the different illustration intensities are plotted in **Figure** **4**a and S4 (Supporting Information). Compared to those of the *I*_DS_–*V*_DS_ curves without a gate voltage (Figure 3c), note that the independence of *I*_DS_ on a negative *V*_DS_ while a strong tunable ability of *I*_DS_ with a positive *V*_DS_ is demonstrated, which indicates the increase of the built‐in potential in Te/WSe_2_ MDHJs originated from the transformation of a p^+^p heterojunction to a p^+^n one. This is due to the fact of shielding effect of Te with high hole concentration and tunability of the work function of the bipolar WSe_2_. Therefore, the *I*_ph_ significantly increases by a positive *V*_GS_ of 50 V owing to the suppression of dark current, which would avail the improvement of the response performance. The linearity power exponent *k* values are further determined to be 1.00, 0.93, and 0.48 when *V*_DS_ is −5, 0, and 5 V, respectively (Figure 4b). The linearity is optimized by applying a *V*_GS_ of 50 V, and the device behaves as an ideal photodetector when *V*_DS_ is −5 V at the PV operation mode.^[^ ^31^ ^]^ However, the *k* value of the device working in the PC mode at *V*_DS_ = 5 V is dropped from 0.61 to 0.48 much lower than the ideal value of 1, which may provide evidence that the response mechanism is mainly dominated by the photogating effect because of the imperfect interface of Te and WSe_2_.^[^ ^34^ ^]^

Moreover, the photoelectric performance of the Te/WSe_2_ MDHJs PDs is further detailed at the positive *V*_GS_ of 50 V. The gate‐tunable power density dependence of the *R* and D* for *V*_DS_ from −5 to 5 V is summarized in Figures 4c and S5 (Supporting Information). Compared to those without a *V*_GS_, note that the values of *R* and D* of the Te/WSe_2_ PDs are not improved significantly when working in the PV mode. However, enhanced response parameters of *R* and D* are obtained for the Te/WSe_2_ MDHJs PDs by applying a positive *V*_GS_ of 50 V when operating in the PC mode. As a result, the peak values of *R* and D* are improved from 27.5 mA W^−1^ and 8.43 × 10^10^ Jones to 13.9 A W^−1^ and 1.37 × 10^12^ Jones with more than 400 and 16 times multiplication, respectively, at a light power density of 0.02 mW cm^−2^.

830 and 1064 nm lasers are also selected to study the photoresponse range of the Te/WSe_2_ PDs. Figure S6 (Supporting Information) shows the detection characteristics under an 830 nm laser illumination. Similar photoelectric behaviors to those of the 638 nm are demonstrated. The maximum values of *R* and D* are decreased to 50.8 mA W^−1^ and 6.9 × 10^9^ Jones when *V*_GS_ is 50 V and *V*_DS_ is 5 V. For a 1064 nm laser illumination, the PV response of the Te/WSe_2_ PDs is absent and only the PC response is observed when *V*_GS_ is 50 V (Figure 4d). The linearity of the *I*_ph_ dependence on *P* is yielded to be 0.67, implying a similar mechanism to that of the 638 nm illumination response (Figure 4e). Extremely weak response with *R* and D* of only 0.15 mA W^−1^ and 1.6 × 10^7^ Jones is found for the 1064 nm laser illumination (Figure 4f).

**Figure 5:**
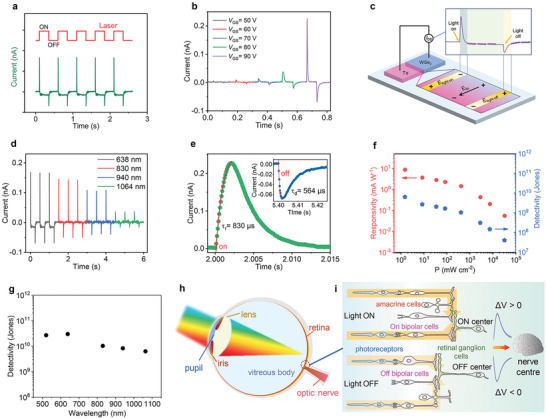
Transient photocurrent behaviors. a) Real‐time output current (green line) via laser pulse (red line). b) Time‐resolved photoresponse spectra at various gates from 50 to 90 V. c) Schematic diagram of the photocurrent induced by *E*_bi_, *E*_Light‐on,_ and *E*_Light‐off_. d) Time‐resolved photoresponse spectra under illumination with the representative wavelengths of 638, 830, 940, and 1064 nm when *V*_GS_ = 90 V. e) Rise time and fall (inset) time of the PTE current. f) Power dependence of *R* and *D* ^*^ induced by PTE effect under 1064 nm laser illumination. g) Peak values of the *D* ^*^ working in PTE mode at different wavelengths. h) Anatomy of the human eye, mainly consisting of the pupil, iris, lens, vitreous body, and retina. i) Two kinds of phototransduction processes (on and off) from the retina to the nerve center.

Intriguingly, abnormal photo‐generated currents (two transient photocurrent signals in opposite directions with positive and negative values) are observed when the light is turning on and off, respectively (**Figure** **5**a) when *V*_GS_ = 50 V. Moreover, this transient photogenerated carrier behavior is present only in the case where a larger positive gate voltage is applied. This phenomenon can be further amplified by increasing the *V*_GS_ (Figure 5b). The peak value of the current is increased from 0.006 to 0.227 nA with ≈38 times increment as the *V*_GS_ is from 50 to 90 V. These data indicate that the PTE effect might be present in the Te/WSe_2_ MDHJs PDs which is caused by the introduction of *V*_GS_.^[^ ^7b^ ^]^ The appearance of PTE is correlated to the construction of the asymmetric Te/WSe_2_ heterojunction device, and the larger thermoelectric figure of merit and adjustability of WSe_2_ with its conduction type and carrier concentration.

When a light beam is turning on or off, the photo‐induced temperature gradient is formed at the interface of the asymmetric Te/WSe_2 heterostructure_ due to their different thermoelectric conversion efficiency of Te and WSe_2_, and thus extrinsic electric fields denoted as *E*_Light‐on_ and *E*_Light‐off_ are yielded by the thermoelectric effect. These two *E*_Light‐on_ and *E*_Light‐off_ will be absent with the disappearance of the temperature gradient when the device is in a stable exposure state. The two opposite transient photocurrents of the Te/WSe_2_ MDHJs PDs are defined as the PTE currents. Note that the two PTE currents show an opposite direction to or the same one as the *E*_bi_ one. As a result, the competition or cooperation between *E*_Light‐on_/*E*_Light‐off_ and *E*_bi_ will be present as schematically displayed in Figure 5c. The relationship of PV and PTE effects regulated by a positive *V*_GS_ is detailed as follows. When a positive *V*_GS_ is applied, the band offset of Te and WSe_2_ decreases, which would lead to the drop of the *E*_bi_ and thus the PV effect weakens.

As for the PTE effect, the thermoelectric conversion efficiency is a crucial parameter and is governed as follows: *zT* = *S* ^2^σ / (*K*_L_ + *K*_e_), in which *S* is the Seebeck coefficient, σ is the electrical conductivity of the materials, *K*_L_ and *K*_e_ are lattice and electronic components of the thermal conductivity, respectively.^[^ ^35^ ^]^ The *S* and σ values are strongly correlated to the band structures and E_F_.^[^ ^35^ ^]^ The positive *V*_GS_ leads to an upward shift of the E_F_ of WSe_2_ while the E_F_ of Te is impervious. Both the absolute values of *S* and *z*T of n‐type WSe_2_ are much larger than that of p‐type WSe_2_.^[^ ^36^ ^]^ In addition, the PTE photocurrent (*I*_PTE_) is defined as $I_{\mathrm{PTE}}=\int_{0}^{x}S\left(x\right)\frac{dT}{dx}\;,$ where *T* is the temperature and *x* represents the position in the heterojunction.^[^ ^37^ ^]^ Hence, when the Te/WSe_2_ MDHJs PDs are tuned to be a p–n heterojunction by a positive *V*_GS_, the PTE effect is improved because of the larger *S* value of WSe_2_ and the *E*_Light‐on_ and *E*_Light‐off_ induced by temperature gradient, and PTE dominates the currents instead of the PV effect, which is further enhanced with increasing the positive *V*_GS_ and also implies that the PTE current occur in the p–n heterojunction rather than in the p^+^p one.

Additionally, the transient photogenerated current behavior is also found at different wavelengths with a positive *V*_GS_ (Figure 5d), and the PTE currents are enhanced with the increase of the incident power at the different wavelengths (Figure S7, Supporting Information). Figure 5e plots the rise time (light turning on) and fall time (light turning off) of the PTE current under the illumination of a 1064 nm wavelength incident laser at the power density of 16.3 W cm^−2^. The photocurrent increases to a peak state shortly and declines to a steady state with a relatively slow decay. The rise time and fall time are well described by a single exponent as indicated by the solid lines in Figure 5e and calculated to be 830 and 564 µs, respectively, showing an order of magnitude faster than those operating at only PV mode (Figure 3e). To better evaluate the response properties both at the cooperation of PTE and PV modes, the peak *R* and D* are up to 8.74 mA W^−1^ and 6.27 × 10^9^ Jones with a power density of 1.50 mW cm^−2^ under a 1064 nm light illumination (Figure 5f). These values are far larger than 0.15 mA W^−1^ and 1.6 × 10^7^ Jones at PV mode, suggesting the improved detection ability by applying a larger positive *V*_GS_ originated from the involvement of the PTE field induced by the existence of temperature gradient at the interface of WSe_2_ and Te. The peak value of D* of the Te/WSe_2_ PDs working in PTE mode at different wavelengths is summarized in Figure 5g. It is found that the PTE response is almost independent of the incident wavelength.

These superior transient optoelectronic performances not only avail the function application in traditional PDs but also expand its function field in bionic vision systems. The basic human visual nervous system mainly consists of a light refraction system, retina, optic nerve, and the visual center of the brain.^[^ ^38^ ^]^ Figure 5g,h describes the anatomy of the human eye and the phototransduction process from the retina to the optic nerve, respectively. The abundant sensory neurons such as photoreceptor cells, amacrines cells, bipolar cells, and retinal ganglion cells are distributed in different layers of the retina and connected by synapses. The phototransduction process will work when the retina is stimulated by optical pulses from external objects.^[^ ^39^ ^]^ The photoreceptors convert the optical signals into electrical signals and trigger the release of neurotransmitters to bipolar cells. Following the photoreceptors, the on/off signals are distinguished by two kinds of bipolar cells through hyperpolarization and depolarization and are further transmitted to the retinal ganglion cells where an action potential is generated and sent to the nerve center through the optic nerve.^[^ ^39^ ^]^ Afterward, the central nervous system regulates the light sensitivity of the eyes through a feedback system. When exposed to an increasing or decreasing light beam, the eyes would initially feel dazzling or dim, and adapt to the changing circumstances immediately with the visual feedback function. In addition, Figure S8 (Supporting Information) shows the PTE response bandwidth of our Te/WSe_2_ phototransistors, and the 3 dB bandwidth is calculated to be 32 Hz. It is worth mentioning that our Te/WSe_2_ devices with transient positive and negative currents from the PTE effects can well simulate the output signals in the visual adaption system, and the response time is also comparable with the reaction speed of human vision.^[^ ^40^ ^]^ Specifically, moreover, the Te/WSe_2_ devices can output a variable transient current by changing the light intensity. These results can well imitate the action potential signal in the visual system and indicate a new function prospect of the Te/WSe_2_ PDs in bionic vision transmission.

Multi‐mechanisms photoresponse such as PC, PV, and PTE modulated by a *V*_GS_ and/or *V*_DS_ is demonstrated in the Te/WSe_2_ MDHJs PDs by the device structure design with the high‐concentration p‐type Te and the bipolar nature of WSe_2_. The boosted device performance and possible working modes are summarized in Table S1 (Supporting Information). The PC effect is responsible for the optoelectronic conversion in the visible band whereas the PTE effect shows a stable and high detectivity in the order of 10^10^ Jones at all these wavelengths and dominates the response in the near‐infrared region. To further assess the performance of the Te/WSe_2_ MDHJs PDs, we compare the crucial parameters such as rectification ratio, responsivity, detectivity, and response time of our device to other Te‐based heterojunctions and PTE PDs as detailed in **Table** **1**. It is clear that the overall device performance of the Te/WSe_2_ MDHJs PDs is much superior to those of these PDs.

**Table 1:** Performance parameters comparison of the Te/WSe_2_ MDHJs PDs and other Te‐based heterojunctions and PTE PDs.

Sample	RR	λ [nm]	*R* [mA W^−1^]	D* [Jones]	*τ*_rising_/*τ*_decay_	Ref.
Te/WSe_2_ PC		638	1.39 × 10^4^	1.37 × 10^12^	1.99 ms /2.64 ms	This work
Te/WSe_2_ PTE	≈10^6^	830	50.8	6.9 × 10^9^	830 µs/564 µs	
		1064	8.74	6.27 × 10^9^		
Te/TiO_2_		350	84	3.7 × 10^9^	0.772 s/1.492 s	[33]
Te/ZnO		300	387	4 × 10^10^	2.46 s/1.75 s	
Te/ReS_2_		632	1.8 × 10^5^	5 × 10^9^	5 ms/8 ms	[10]
Te/MoTe_2_	1310	940	24.7	4.8 × 10^7^	4.8 ms/24.6 ms	[26]
Te/CdS	330	980	0.02	5 × 10^7^	0.46 s/0.5 s	[39]
Te/Graphene		637	6	5 × 10^8^		[43]
Te/In_2_S_3_	≈10^4^	635	2.75	1.7 × 10^9^	0.48 s/0.52 s	[12]
Te/WS_2_		635	3.69 × 10^3^	1.34 × 10^14^	25 ms/14.7 ms	[28]
Te/Bi_2_Se_3_		700	0.17	1.09 × 10^9^	0.01 s/0.07 s	[27]
Te/Bi		700	0.14	1.2 × 10^8^	0.05 s	[44]
Te/CsPbBr_3_		500	0.35	1.42 × 10^10^	0.09 ms	[32]
The flakes		1000	10^3^	2 × 10^9^		[41]
Bi_2_Se_3_/GaN		800	0.6		66 ms/43 ms	[5]
InSe/Si	5000	400	934	2.1 × 10^12^	32 ms/60 ms	[3a]
PdSe_2_/CdTe	≈2 × 10^4^	780	324	3.3 × 10^12^	4.9 µs/8.3 µs	[3b]
MoTe_2_/MoS_2_	80	637	43.6	1.06 × 10^2^	0.06 ms	[6a]
SnSe PTE		532		2 × 10^9^	7.2 ms	[7a]
WSe_2_ PTE		500	0.7		≈10 ms	[16]

### 2.4 Analysis of Photoelectrical Mechanisms of the Te/WSe_2_ MDHJs PDs

**Figure 6:**
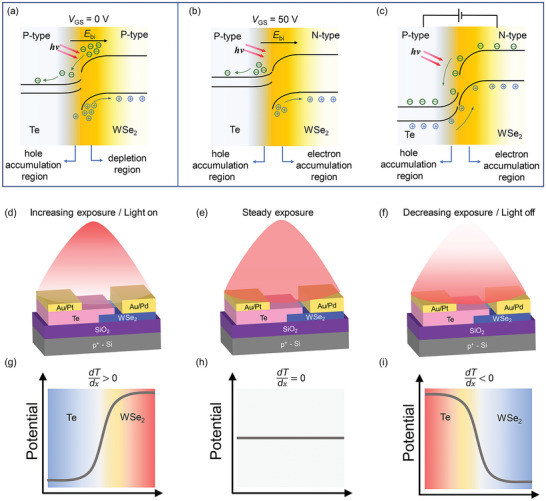
Possible response mechanisms. a, b) Energy band diagram of the Te/WSe_2_ heterojunction under illumination at zero bias when *V*_GS_ = 0 V and *V*_GS_ = 50 V, respectively. c Energy band diagram of the Te/WSe_2_ heterojunction at a large positive bias. d–f) Schematic diagrams of the device working under increased exposure/light on, steady exposure, and decreased exposure/light off conditions, respectively. g–i) The spatial distribution of the PTE potential in Te/WSe_2_ heterojunction corresponding to the cases in Figure 6d–f.

To clarify the possible origins of the multi‐mechanism effects, transient photocurrent, and improved response performance of the Te/WSe_2_ MDHJs PDs, the evolution of the energy band with the applied gate and bias voltages is schematically plotted in **Figure** **6**. Typical type I homogenous heterojunction is formed as expected with p‐type Te with high hole concentration and weak p‐type WSe_2_, the Te/WSe_2_ without a gate or a bias (Figure 6a). When the two semiconductors are contacted, the E_F_ of the narrow bandgap heavily doped Te is much higher than that of WSe_2_, and thus the holes in the WSe_2_ side would flow to the Te one to reach a thermal equilibrium state. As a result, a hole‐accumulated region is present in Te while a unilateral depletion one occurs in WSe_2_ and thus a p^+^p junction is formed at the interface, which leads to an *E*_bi_ pointing from Te to WSe_2_. With light illumination, the photon‐generated carriers are easily separated at the depletion region of WSe_2_ with the aid of the *E*_bi_. Specifically, the photo‐induced electrons in the conduction band of WSe_2_ can directly cross the junction without any block of the barrier while the holes in Te are accumulated and recombined with these drifting electrons. In this case, the probability of the carrier recombination in the WSe_2_ depletion region decreases and the photogenerated holes are responsible for the photocurrent. When a positive *V*_GS_ is applied, the E_F_ of Te is almost independent of the *V*_GS_ whereas the E_F_ of WSe_2_ shifts upward, leading to a transformation from a p^+^p junction to a p–n one. Note that the E_F_ of p‐type Te is still higher than that of WSe_2_ and the *E*_bi_ is still from the Te side to the WSe_2_ one (Figure 6b). This can bring about the increase of the hole barrier and thus a huge increase of the *V*_T_ (Figure 2b). When a large forward bias is also applied, the Te/WSe_2_ heterojunction is proposed to be a type II p–n junction (Figure 6c), and the transport of the electrons and holes is dominated by the drift–diffusion mechanism.^[^ ^4^, ^12^ ^]^ The applied electric field further accelerates the flow of the photon‐generated carriers in depleted regions to the electrodes and thus enhances the response performance of the Te/WSe_2_ MDHJs PDs.

Figure 6d–i represents the PTE response dynamic with light illumination, including i) an increasing exposure/light‐on process, ii) a steady exposure state, and iii) a decreasing exposure/light‐off process. When the light intensity is increasing, the photothermal effect occurs ineluctably due to the large thermoelectric figure of merit of WSe_2_ through a gate modulation and is further enhanced with the increasing incident power.^[^ ^36^ ^]^ In this condition, a spatial thermal gradient (*dT/dx* > 0) is generated and the PTE potential field direction in heterojunction is from WSe_2_ to Te (Figure 6d,g). Hence, a sharp positive output signal (ΔV > 0) is observed and decays gradually as the thermal gradient disappears. At the (ii) stage, the exposure is steady without thermal gradient in heterojunction. In this case, there is no extra electric field (Figure 6e,h). Subsequently, an inverse thermal gradient (*dT/dx* < 0) is formed at the (iii) condition, and a negative output signal (ΔV < 0) is demonstrated (Figure 6f,i). These transient positive and negative output signals can be regarded as the on bipolar cells that transmit action potential signals and the off bipolar cells output, respectively. As a consequence, the Te/WSe_2_ MDHJs can take responsibility for a part of the artificial bionic visual adaptation system to generate and transmit variational visual information, effectively protect the human eye to the complex environment, and especially reduce the damage by short‐wavelength infrared light on the retina and lens.^[^ ^38b^ ^]^ These results including excellent response capabilities in visible to near‐infrared regions and the simulated excitatory or inhibitory postsynaptic currents transmission behaviors indicate that the Te/WSe_2_ MDHJs are the candidate for the advanced optoelectronic sensors and artificial bionic visual adaptation systems.

## 3 Conclusion

In summary, we fabricate Te/WSe_2_ MDHJs phototransistors by a vdW integration route. A low dark current of less than 0.1 pA as well as a large on/off rectification ratio of 10^6^ is obtained in Te/WSe_2_ MDHJs phototransistors. Electrically tunable multi‐mechanism synergistic coupling including PC, PV, and PTE effects significantly boost the *R* and *D* ^*^, and their peak values can reach 13.9 A W^−1^ and 1.37 × 10^12^ Jones, respectively, exceeding 400 times increase. The presence of the transient positive and negative photocurrent signals and comparable response time of 830 µs to that of the human visual system suggests that Te/WSe_2_ MDHJs PDs can well imitate the function of the human visual system.

## 4 Experimental Section

### Fabrication

The mechanically exfoliated WSe_2_ flakes from bulk single crystals were transferred on a p‐type heavily doped Si substrate covered with a 300 nm thick SiO_2_. 65 nm thick single‐crystalline Te thin films were grown by MBE (Riber 32P) on mica van der Waals substrates along [001] direction. Then, a PDMS film was covered on the Te thin films separated Te films from the mica substrate, and transferred to the WSe_2_/SiO_2_/Si flakes. The electrodes were patterned by electron beam lithography (FEI Inspect F50 SEM). Then, the Pt/Au metal (25 nm/75 nm) and Pd/Au metal (15 nm/45 nm) electrodes were deposited on Te and WSe_2_, respectively, through thermal evaporation deposition (JSD‐300).

### Characterization and Measurement

The morphology structures and thicknesses of Te and WSe_2_ were measured by AFM. The Raman data were collected on a Renishaw inVi Reflex Raman microscope with a 532 nm excitation wavelength. A cross‐sectional Te/WSe_2_/SiO_2_ sample was prepared using a focused ion beam (FEI Helios G4 UX). The HRTEM and EDS mapping were conducted using a JEOL JEM‐ARM300 to obtain the interface information. Electronic and optoelectronic measurements were measured using an MStarter200 semiconductor characterization system integrating a Keithley 2450 and 6482 respectively. Response time was recorded using a PicoScope 4262 oscilloscope. The picoammeter 6482 was connected to the metal electrodes as a voltage source. The gate voltage by a sourcemeter 2450 was applied to the Si substrate, and the device drain was grounded. The parameters of the PC working mode were calculated by extracting the photocurrents in output curves. The parameters of PV and PTE working modes were calculated by the time‐resolved photocurrent spectra. The light power was controlled by changing the output voltage of the laser controller. The parameters of PC response were calculated by extracting the photocurrents in output curves at their corresponding bias voltages. The parameters of PV and PTE response were calculated by the time‐resolved photocurrent spectra.

## Conflict of Interest

The authors declare no conflict of interest.

## Author Contributions

H.C., T.H., Y.C., and W.B. conceived and supervised the research. H.C. and T.H. fabricated the devices. T.H., D.Z., X.Z., and X.W. performed the structural measurement. H.C., T.H., and J.Z. performed the electrical and optoelectronic measurements. J.Y., Y.Z., X.T., H.S., J.W., and J.C. advised on the experiments and data analysis. H.C. and W.B. co‐wrote the paper. All authors discussed the results and revised the manuscript.

## Supporting information

Supporting Information

## Data Availability

The data that support the findings of this study are available from the corresponding author upon reasonable request.
