# Supplementary material for: Electrically Tunable Multiple‐Effects Synergistic and Boosted Photoelectric Performance in Te/WSe2 Mixed‐Dimensional Heterojunction Phototransistors
Source: Adv Sci (Weinh). 2024 Mar 19;11(22):2400018. doi: 10.1002/advs.202400018 (PMC11165519; doi:10.1002/advs.202400018)
Supplement: Supplementary file 1 — Supporting Information [file ADVS-11-2400018-s001.pdf]

ADVANCED SCIENCE
Open Access

Supporting Information

for *Adv. Sci.*, DOI 10.1002/advs.202400018

Electrically Tunable Multiple-Effects Synergistic and Boosted Photoelectric Performance in Te/WSe_2 Mixed-Dimensional Heterojunction Phototransistors

Hechun Cao, Tao Hu, Jiyue Zhang, Dongyang Zhao, Yan Chen, Xudong Wang, Jing Yang, Yuanyuan Zhang, Xiaodong Tang*, Wei Bai*, Hong Shen, Jianlu Wang and Junhao Chu*

Supplementary

Electrically Tunable Multiple-effects Synergistic and Boosted Photoelectric Performance in Te/WSe_2 Mixed-dimensional Heterojunction Phototransistors

Hechun Cao[1,2,#], Tao Hu[1,2,#], Jiyue Zhang, [1] Dongyang Zhao[1,2], Yan Chen[2,3*], Xudong Wang[2], Jing Yang[1], Yuanyuan Zhang[1], Xiaodong Tang[1,4*], Wei Bai[1*], Hong Shen[2], Jianlu Wang[2,3,5], and Junhao Chu[2,3]

H. Cao, T. Hu, J. Zhang, D. Zhao, J. Yang, Y. Zhang, X. Tang, W. Bai
Key Laboratory of Polar Materials and Devices (MOE) and Department of Electronics, East China Normal University, Shanghai 200241, P.R. China
Email: xdtang@sist.ecnu.edu.cn; wbai@mail.sitp.ac.cn

H. Cao, T. Hu, D. Zhao, Y. Chen, X. Wang, H. Shen, J. Wang, J. Chu
State Key Laboratory of Infrared Physics, Shanghai Institute of Technical Physics, Chinese Academy of Sciences, No.500 Yutian Road, Shanghai 200083, China.
Email: yanchen_@fudan.edu.cn

Y. Chen, J. Wang, J. Chu
Shanghai Frontier Base of Intelligent Optoelectronics and Perception, Institute of Optoelectronics, Fudan University, 200433 Shanghai, China.

X. Tang
Collaborative Innovation Center of Extreme Optics, Shanxi University, Taiyuan, Shanxi 030006, P. R. China

J. Wang
Frontier Institute of Chip and System, Fudan University, Shanghai 200433, China

Contents:

I. Growth and Characterization of Te thin films grown by MBE

II. Transfer characteristics of devices.

III. Optoelectronic measurement of Te/WSe_2 heterojunction devices

I. Growth and Characterization of Te thin films grown by MBE

The Te thin films were grown by MBE on mica (001) with an ultrahigh vacuum background pressure of 1×10^{-10} Torr. Te (99.9999%) solid source was evaporated from Knudsen cell at 350 ℃ for 20 minutes and the substrate is heated at 220℃. In situ reflection high-energy electron diffraction (RHEED, STAIB Instruments NEK 300R3-8) with a voltage of 20 kV and current of 1.6 A was applied to monitor the whole growth processes. The RHEED result shows it highly oriented single crystal. The high-resolution X-ray diffraction (HRXRD) spectrum as well reciprocal space mapping (RSM) show that Te (003) peak in addition to mica (00*l*) peaks, which indicating a substantially strong preference for the Te epi-film from the out-of-plane (001) orientation. The value of full width half medium (FWHM) of rocking curve orienting Te (003) achieves 1.1°, and the Phi scanning indicates six-degrees of symmetry of the Te epi-film. The Scanning electron microscopy (SEM) shows that the Te epi-films have good densification. These data are given in Figure S1.

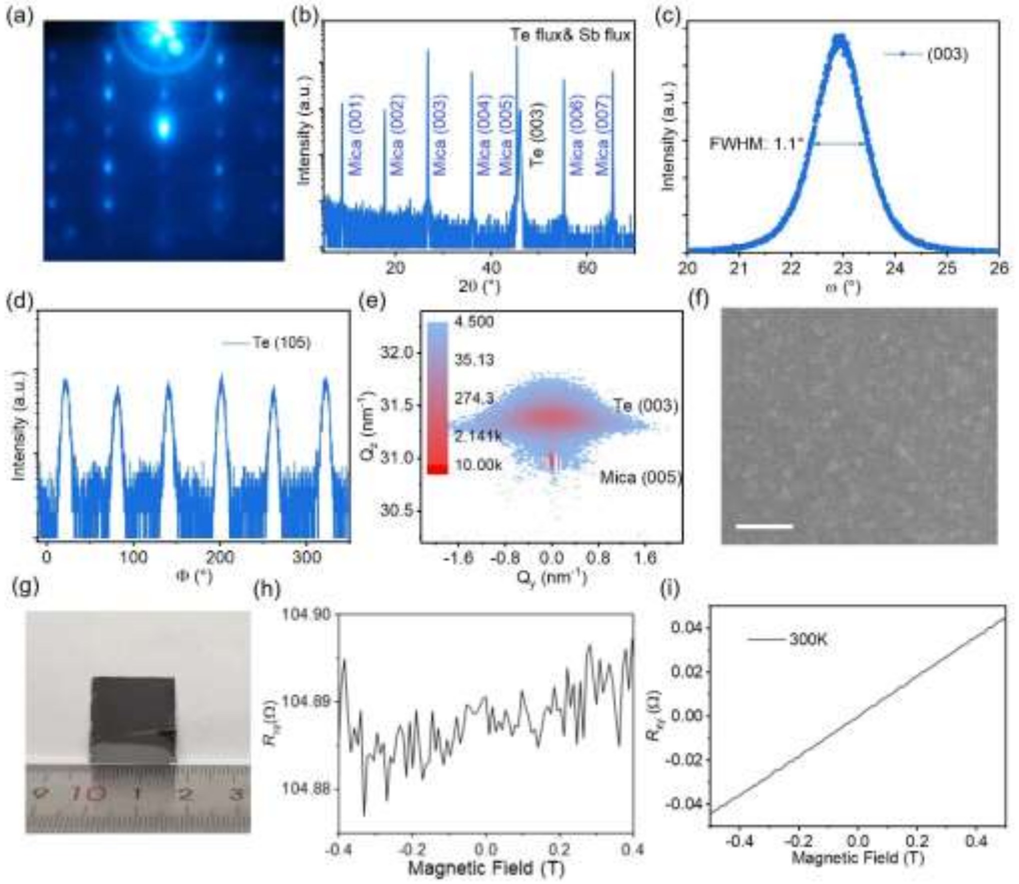

Figure S1. Structural characterization of MBE growth Te thin films. a) RHEED patterns of Te thin films after growth. b) HRXRD 2θ-ω diffraction patterns of Te/Mica. c) Rocking curve of Te (003) diffraction peak. d) Phi-scan along Te (105) direction. e) The asymmetric RSM of Te/mica. f) SEM images of the surface of Te thin films. The scale bar is 1 μm. g) Optical image of Te thin films. h) and i) Hall measurement result of R_{xx} and R_{xy}, showing carrier density of 7×10^{18} cm-3 and mobility of 21 $cm^{-2}V^{-1}s^{-1}$ at 300 K.

II. Transfer characteristics of devices.

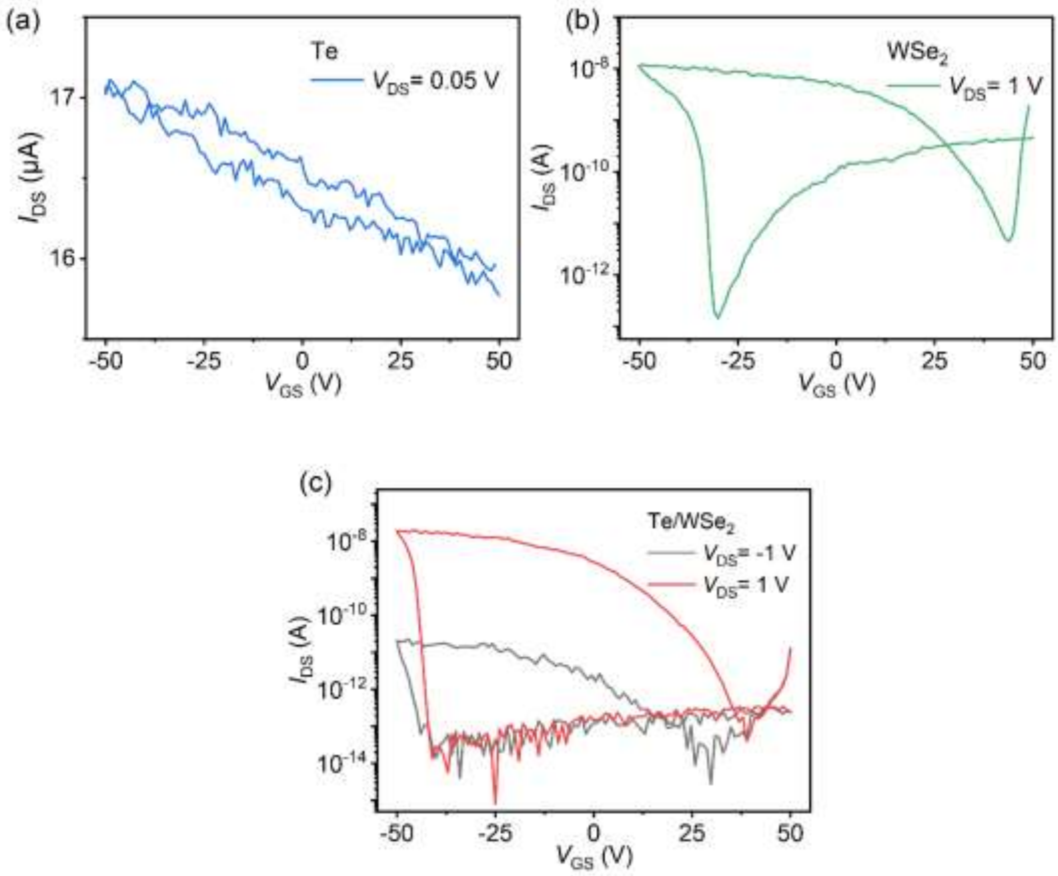

Figure S2. Transfer characteristics of Te (a), WSe_2 (b), and Te/WSe_2 heterojunction (c) devices.

Figure S2 shows the p-type conduction characteristic of Te whereas WSe_2 is an ambipolar semiconductors. And the ambipolar WSe_2 plays as a dominate role in the Te/WSe_2 heterojunction. When applying a large V_{GS}, the Te/WSe_2 heterojunction would transfer from p-p type to p-n type.

III. Optoelectronic measurement of Te/WSe_2 heterojunction devices

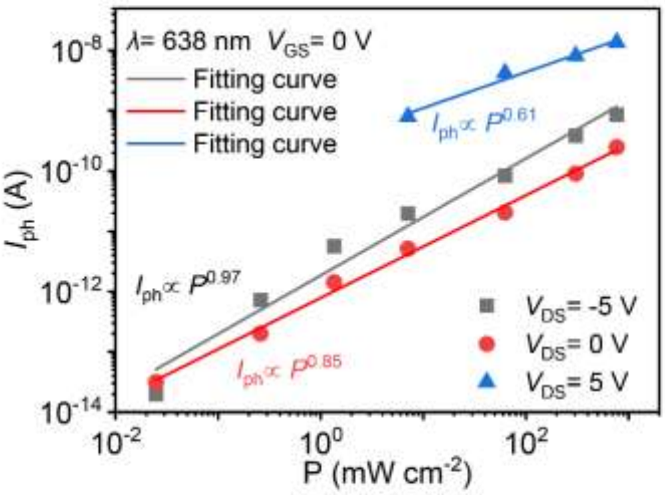

Figure S3. Power dependence of I_{ph} at V_{DS} = -5 V, 0 V and 5 V without gate voltage.

The light intensity dependence on the photocurrent is present in Figure S3 for V_{GS} = 0 V. The fitting exponent k values are 0.85, 0.97 and 0.61 with V_{DS} of -5 V, 0 V and 5 V, respectively.

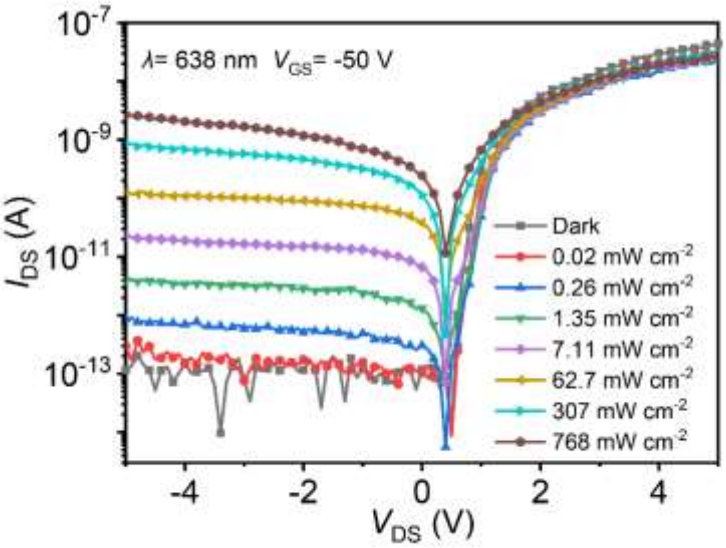

Figure S4. Output curves in the dark case and under a 638 nm laser illumination with various power densities at V_{GS} = -50 V.

I_{DS}-V_{DS} curves at V_{GS} = -50 V for various illustration intensities are plotted in Figure S4. In this case, the photocurrent only remains at zero bias or a negative V_{DS}.

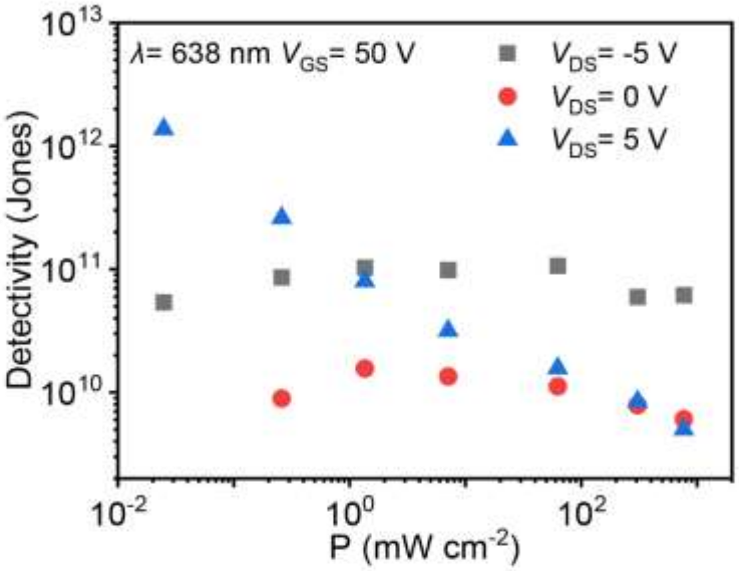

Figure S5. Power dependence of the Detectivity at V_{DS} = -5 V, 0 V and 5 V when V_{GS} at 50 V.

Figure S5 shows the power dependence of the $D*$ at V_{GS} = 50 V, and the peak value is 1.37×10^{12} Jones at a light power density of 0.02 mW cm^{-2} when V_{DS} = -5 V.

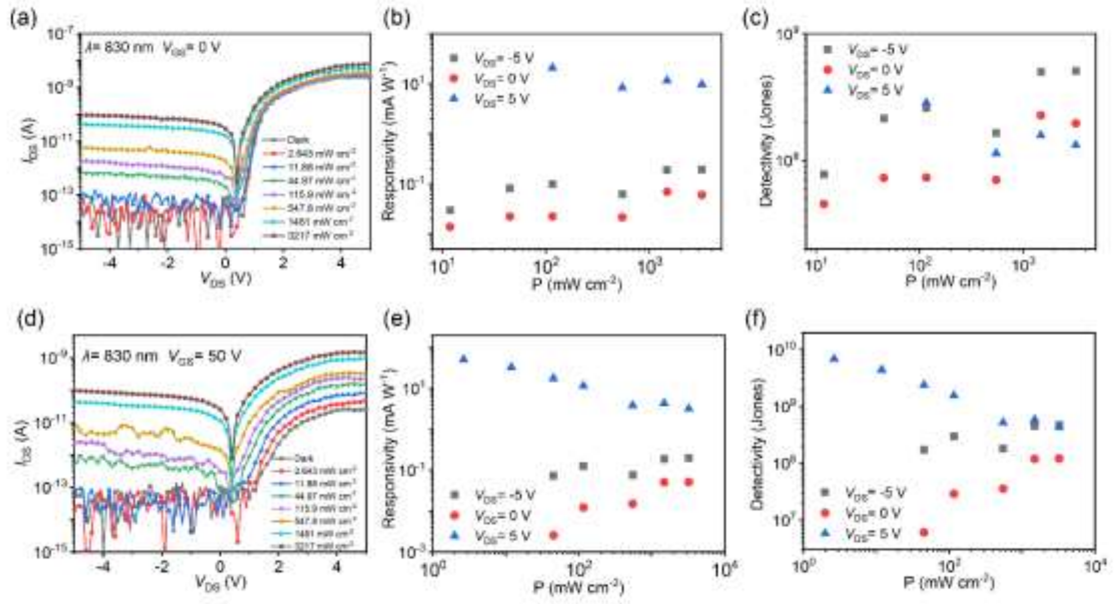

Figure S6. Optoelectronic properties of the Te/WSe_2 vdWH PDs under a 830 nm laser. Output curves with various power densities (a), Power dependence of *R* (b), and *D** (c) at V_{GS} = 0 V. Output curves with various power densities (d), Power dependence of *R* (e), and *D** (f) at V_{GS} = 50 V.

The detection characteristics under an 830 nm laser illumination and 50 V are present in Figure S6 (a-c) for V_{GS} at 0 V and (d-f) for V_{GS} at 50 V. Here the V_{GS} of 50 V significantly enhances both *R* and *D** when applying a positive V_{DS}.

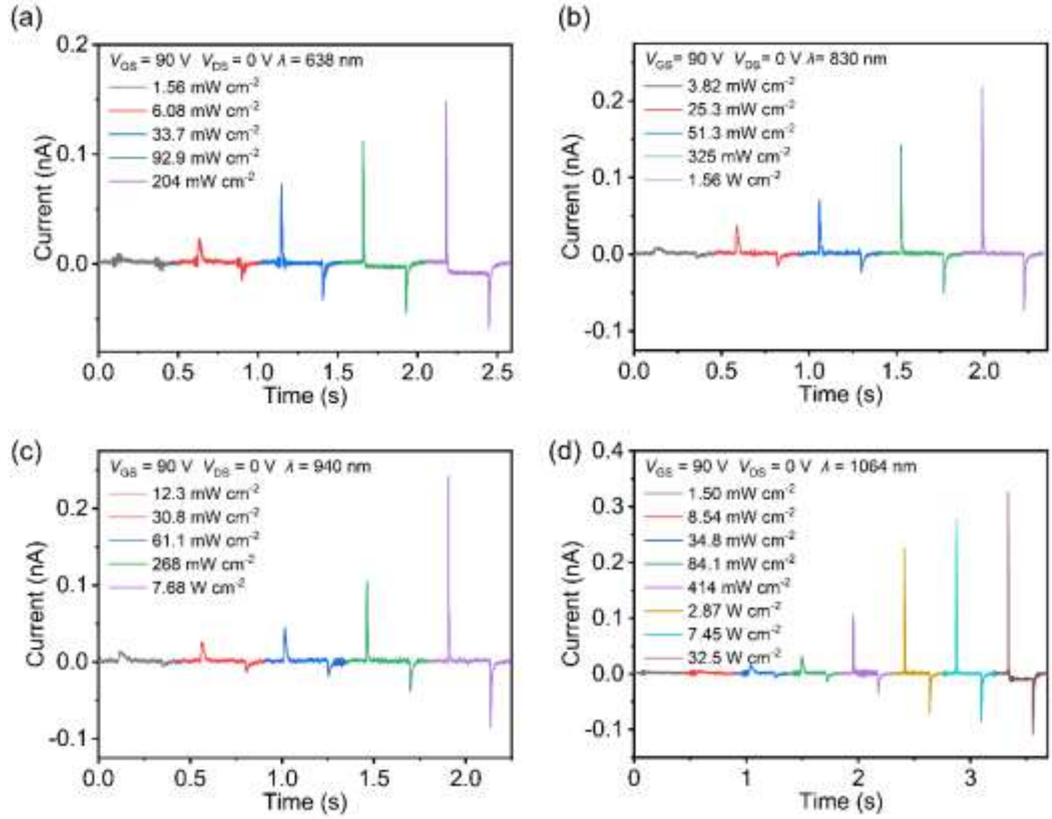

Figure S7. Time-resolved photoresponse spectra of the Te/WSe_2 PDs at various incident power under 638 nm (a), 830 nm (b), 940 nm (c), and 1064 nm (d) illumination when V_{GS} = 90 V.

Figure S7. exhibits the transient PTE response behaviors of Te/WSe_2 PDs from 638nm to 1064 nm, and the PTE current displays obvious power dependence with the increasing incident light intensity.

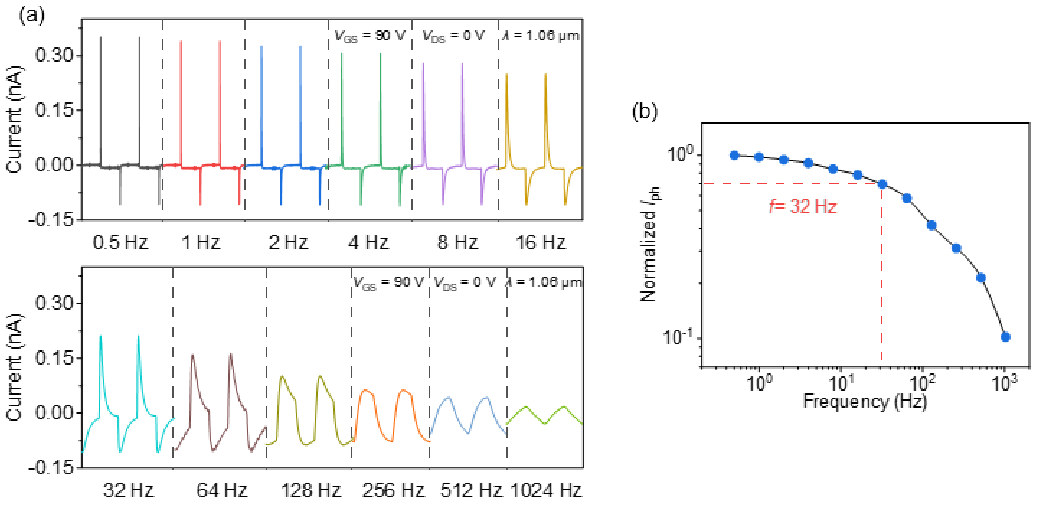

Figure S8. (a) Time-resolved photocurrent spectra of Te/WSe_2 PDs at different frequencies. (b) Normalized photocurrent variation with frequency

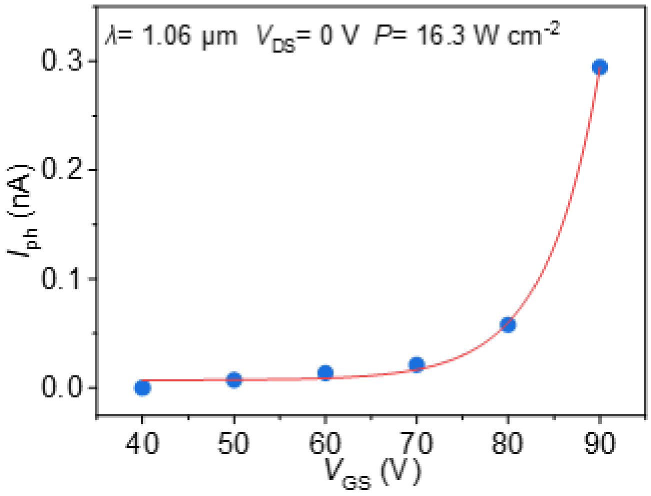

Figure S9. The PTE currents of the device with 1064 nm incident laser under various V_{GS}.

Table S1. Electrical boosted detectivity of Te/WSe_2 PDs and the corresponding working mode at different wavelength.

Wavelength	V_{DS}	V_{GS}	
		> 0 V	0 V

		Working mode & D^*	
638 nm	+5 V	PC; 1.37×10^{12}	PC; 9.23×10^{9}
	0 V	PTE; 3.1×10^{10}	PV; 1.2×10^{10}
830 nm	+5 V	PC; 6.9×10^{9}	PC; 2.8×10^{8}
	0 V	PTE; 1.03×10^{10}	PV; 2.3×10^{8}
1064 nm	+5 V	PC; 1.6×10^{7}	None
	0 V	PTE; 6.27×10^{9}	None

The electrical boosted detectivity of the device and corresponding working mode are summarized in Table S1. The device shows great visible detective ability when working at PC mode ($V_{DS} > 0$ V). The positive V_{GS} extends the response ability of the device for near infrared.
